# Nuclear Translocation of Jacob in Hippocampal Neurons after Stimuli Inducing Long-Term Potentiation but Not Long-Term Depression

**DOI:** 10.1371/journal.pone.0017276

**Published:** 2011-02-18

**Authors:** Thomas Behnisch, PingAn YuanXiang, Philipp Bethge, Suhel Parvez, Ying Chen, Jin Yu, Anna Karpova, Julietta U. Frey, Marina Mikhaylova, Michael R. Kreutz

**Affiliations:** 1 Institutes of Brain Science, Fudan University, Shanghai, China; 2 State Key Laboratory of Medical Neurobiology, Fudan University, Shanghai, China; 3 Project Group Neuroplasticity, Leibniz Institute for Neurobiology, Magdeburg, Germany; 4 Department of Neurophysiology, Leibniz Institute for Neurobiology, Magdeburg, Germany; 5 Department of Toxicology, Hamdard University, New Delhi, India; The Research Center of Neurobiology-Neurophysiology of Marseille, France

## Abstract

**Background:**

In recent years a number of potential synapto-nuclear protein messengers have been characterized that are thought to be involved in plasticity-related gene expression, and that have the capacity of importin- mediated and activity-dependent nuclear import. However, there is a surprising paucity of data showing the nuclear import of such proteins in cellular models of learning and memory. Only recently it was found that the transcription factor cyclic AMP response element binding protein 2 (CREB2) transits to the nucleus during long-term depression (LTD), but not during long-term potentiation (LTP) of synaptic transmission in hippocampal primary neurons. *Jacob* is another messenger that couples NMDA-receptor-activity to nuclear gene expression. We therefore aimed to study whether Jacob accumulates in the nucleus in physiological relevant models of activity-dependent synaptic plasticity.

**Methodology/Principal Findings:**

We have analyzed the dynamics of Jacob's nuclear import following induction of NMDA-receptor dependent LTP or LTD at Schaffer collateral-CA1 synapses in rat hippocampal slices. Using time-lapse imaging of neurons expressing a Jacob-Green-Fluorescent-Protein we found that Jacob rapidly translocates from dendrites to the nucleus already during the tetanization period of LTP, but not after induction of LTD. Immunocytochemical stainings confirmed the nuclear accumulation of endogenous Jacob in comparison to apical dendrites after induction of LTP but not LTD. Complementary findings were obtained after induction of NMDA-receptor dependent chemical LTP and LTD in hippocampal primary neurons. However, in accordance with previous studies, high concentrations of NMDA and glycine as well as specific activation of extrasynaptic NMDA-receptors resembling pathological conditions induce an even more profound increase of nuclear Jacob levels.

**Conclusions/Significance:**

Taken together, these findings suggest that the two major forms of NMDA-receptor dependent synaptic plasticity, LTP and LTD, elicit the transition of different synapto-nuclear messengers albeit in both cases importin-mediated retrograde transport and NMDA-receptor activation is required.

## Introduction

It is generally believed that synapse-to-nucleus communication plays an important role for long-term memory formation [1]–[3]. However, the control of activity-dependent gene expression by synaptic signals is still far from being understood. Besides the transduction of synaptic Ca^2+^-signals via dendritic action potentials or intradendritic Ca^2+^-waves in recent years a growing number of synapto-nuclear protein messengers have been shown to enter the nucleus particularly in response to NMDA-receptor (NMDAR) activation [3]–[9]. Collectively these latter findings lead to the hypothesis that nucleocytoplasmic shuttling of proteins deriving from synapses and dendrites might be directly involved in plasticity-related gene expression and thereby contribute to memory formation [3], [10]. This hypothesis is confronted with a surprising paucity of data showing the nuclear import of synapto-nuclear protein messengers in cellular models of learning and memory like long-term potentiation (LTP) or long-term depression (LTD). LTP and LTD are activity-dependent forms of synaptic plasticity that in the cornu ammonis 1 (CA1) region of the hippocampus require calcium influx through NMDA receptors (NMDARs) [11]. The induction of LTP and LTD at these synapses correlates with learning processes *in vivo* and is thought to underlie memory formation [11]–[13].

Previous work has shown that neuronal importins are present in dendrites and synapses where they can directly associate with NMDARs [14], [15]. Importantly, importin-α and -β traffic from synapses and distal dendrites to the nucleus in a NMDAR-dependent manner [14], [15]. Jacob is a potential messenger molecule on this importin-α/-β pathway to the nucleus since its activity-dependent nuclear accumulation requires binding to importin-α and strictly depends upon NMDA-receptor activation [7], [9], [16]. We therefore undertook the effort to investigate the dynamics of the nuclear import of Jacob during induction of NMDA-receptor dependent LTP and LTD at Schaffer collateral synapses in the CA1 region of the hippocampus.

## Results

### The induction of LTP leads to nuclear translocation of Jacob

**Figure 1:**
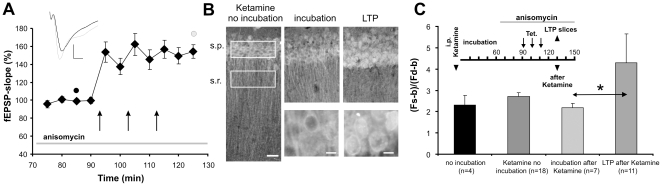
Jacob accumulates in the nucleus after LTP induction. A) The diagram shows averaged normalized fEPSP-slope values over time. LTP was induced by 3 trains of 100 Hz/1-s tetanization (arrows) at 10-min intervals. Slices were collected 40 minutes after the last baseline recording. The fEPSP transients are depicted for the time points as indicated by black and gray filled circles. B) For the conditions ‘Ketamine no incubation’, ‘incubation' and ‘LTP’ representative digital images of Jacob immunofluorescence are presented. Under control conditions Jacob is localized in proximal apical dendrites and is enriched at the nuclear membranes of CA1 neurons. After LTP induction the immunofluorescence in the str. pyramidale (s.p.) is more intense whereas the fluorescence signal is reduced in the str. radiatum (s.r.) area. The white boxes represent region of interests (ROIs) taken for the measurement of averaged intensities within the s.p. and s.r. field. Higher magnification images of s.p. are presented (scale bar: 10 µm). C) The diagram depicts the results of the quantitative analyses of the immunofluorescence under the various conditions. The ratio of s.p. to s.r. was calculated after subtracting background values (b; minimum value in dendritic area) from the averaged fluorescence intensity of s.r. (Fd: dendritic fluorescence) and s.p. (Fs: somatic fluorescence). After LTP induction the ratio is significant larger than the ratio from “incubation after Ketamine” slices. The inlet summarizes the design of the experiment.

We first investigated whether Jacob translocates from dendritic compartments of the stratum radiatum to the nuclei of CA1 neurons after tetanization of Schaffer-collateral inputs. To this end, we performed Jacob immunostainings of acute hippocampal slices from Ketamine anaesthetized animals. Ketamine was applied to reduce the amount of Jacob accumulation in the nucleus due to unspecific activation of synaptic transmission during acute slice preparation. To analyze the relative immunofluorescence we subtracted the background intensity value from the intensity values measured in str. radiatum and str. pyramidale. We found that the intensity ratio of str. pyramidale to str. radiatum changed significantly as compared to control slices following tetanization (**Fig. 1A–C**). Subsequent to the addition of anisomycin to the bath medium, to preclude the possibility that de novo protein synthesis might obscure the result, we found an increase of Jacob in the str. pyramidale within 10 minutes after the last tetanization, as compared to non tetanized hippocampal slices (**Fig. 1A–C**).

### LTP induction evokes translocation of Jacob-GFP

**Figure 2:**
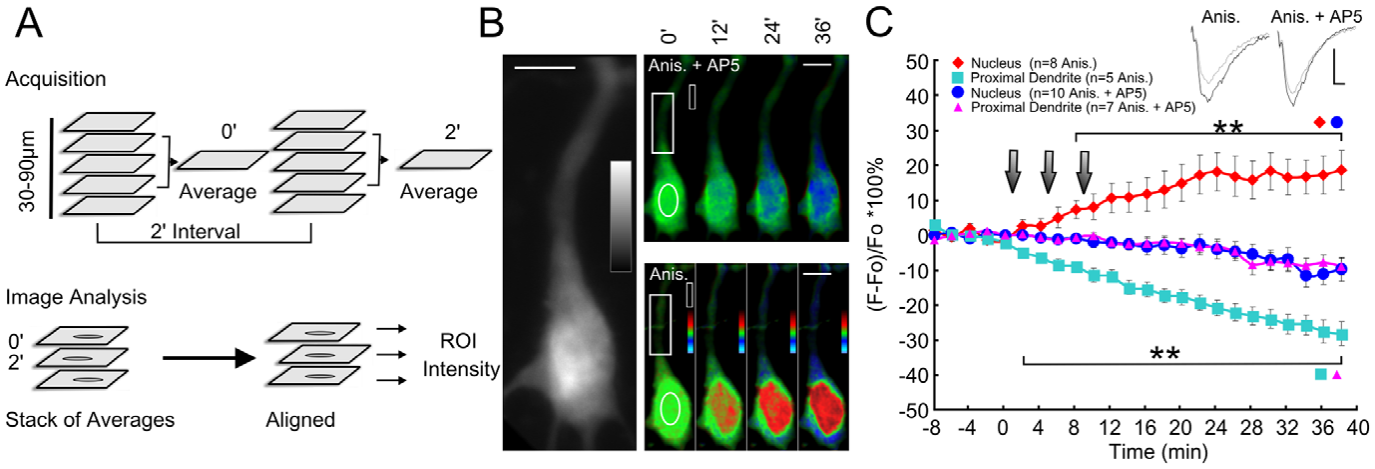
Time-lapse imaging of Jacob-GFP nuclear translocation in acute hippocampal slices in response to LTP induction. A) Schematic representation of data acquisition and image analysis. B) A representative 12 bit grayscale confocal image of a neuron expressing Jacob-eGFP (grayscale range: 0: black to 4095: white). Other images represent F/F_0_ images that were acquired at the indicated time points. ROIs are indicated for nucleus, proximal dendrite and background area with white circles and squares (horizontal white scale bar: 10 µm). The colored vertical bar encodes green as 0, and dark blue as -20% and red as +20%. C) The time course of normalized fluorescence intensities is depicted for nuclei and proximal dendrites acquired under application with anisomycin (Anis., 50 µM) and Anis. + AP5 (40 µM). Brackets and asterisks (**, p≤0.05; Mann-Whitney U-Test) indicate the significance intervals between various groups (symbol pairs on bracket). The inlet shows fEPSP-transients before, and 5 minutes after the first tetanization for Anis. and Anis.+AP5 experiments. Scale bars indicate 0.5 mV for vertical and 2 ms for horizontal bar. Vertical arrows show the time point of 100 Hz/1-s tetanization.

It has been shown previously that the requirement for de novo protein synthesis in order to maintain LTP may have a critical time window [17], [18]. Frey and co-workers found that the blocker of gene transcription actinomycin D was only effective in influencing the maintenance of LTP when applied before tetanization, while it was ineffective when it was administered after tetanization [18]. It can therefore be expected that it is crucial for a synapto-nuclear messenger to be present in the nucleus already during induction of synaptic plasticity in order to have a role in plasticity-related gene transcription. We therefore asked how fast Jacob enters the nucleus during the induction of LTP. For this purpose we infected the CA1 region of hippocampal slices with a Semliki Forest virus expressing full-length Jacob fused to an eGFP-tag. Using this approach we found many cells transduced with Jacob-eGFP reporter construct within 12 hours that exhibited an even distribution of eGFP-fluorescence in dendrites of the str. radiatum, soma and nucleus (**Fig. 2A–B**). Following addition of anisomycin to prevent further protein synthesis, which might have obscured the results, the slices were tetanized and the kinetics of the re-distribution of Jacob was determined with time-lapse confocal microscopy. Subsequent image analysis revealed a significant increase of Jacob-GFP fluorescence in the nucleus within 10 minutes after the last baseline recording (**Fig. 2B–C**). The fluorescence signal in the nucleus increased continuously for 20 minutes and remained stable up to the end of recording (**Fig. 2C**). The translocation of Jacob into the nucleus was accompanied by a decrease of Jacob-GFP fluorescence in proximal dendrites (**Fig. 2B–C**). To confirm that Jacob translocation was specifically related to induction of LTP, we performed similar experiments in the presence of the NMDA-receptor antagonist AP5 (40 µM). AP5 is known to block the induction of Schaffer-collateral-CA1 LTP [19], [20]. Under these conditions no increase of Jacob-GFP fluorescence intensity was detectable in the nucleus (**Fig. 2B–C**). Interestingly, the increase in nuclear Jacob-GFP fluorescence in the LTP (as compared to the AP5-LTP group) became significant from the 8^th^ minute, whereas already from the 2^nd^ minute onwards a significant decrease was found for the proximal dendrites (p<0.05) (**Fig. 2C**).

### The induction of early-LTP does not induce the nuclear accumulation of Jacob

**Figure 3:**
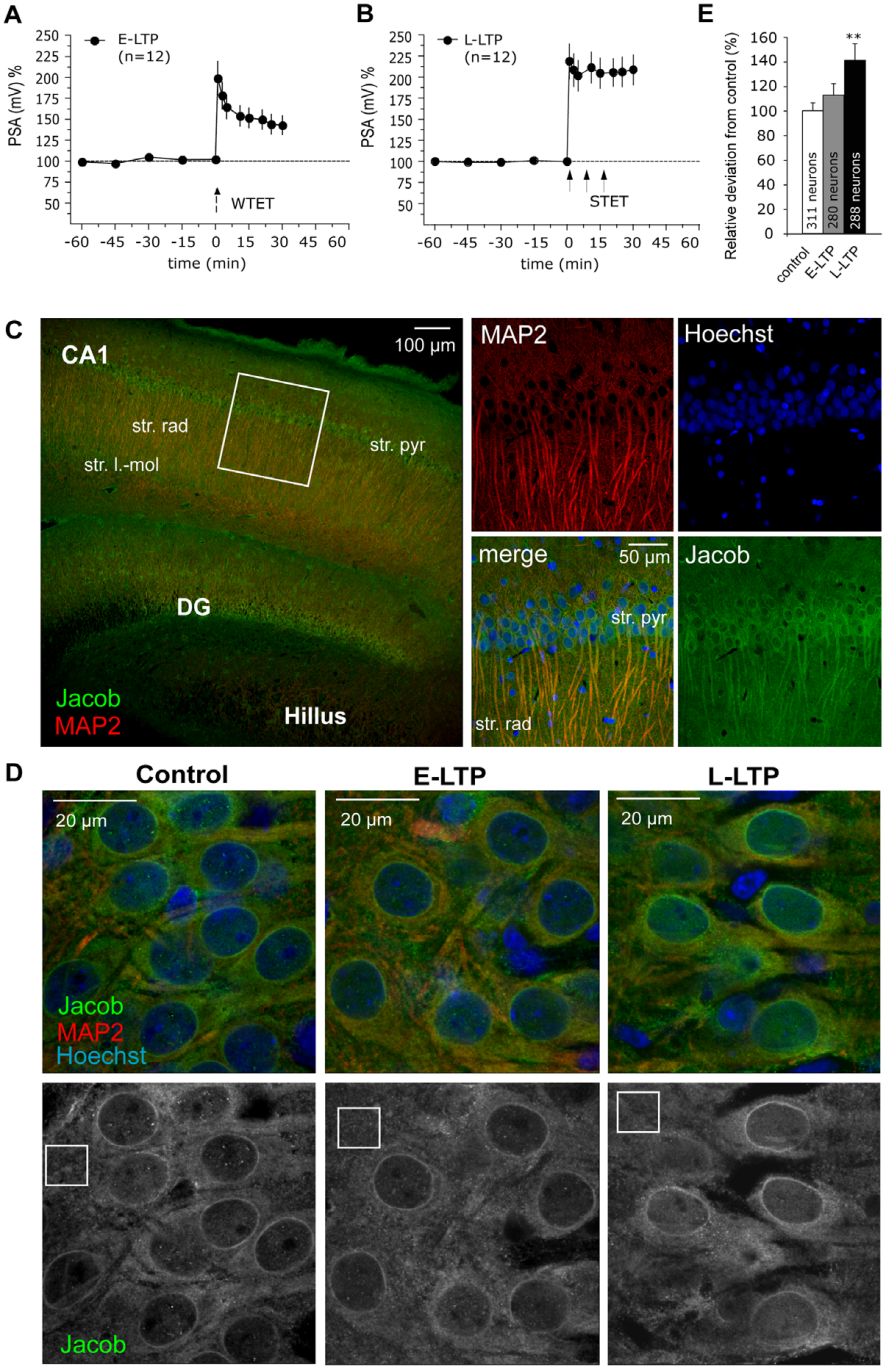
Jacob translocation after induction of early (E)- and late (L)-LTP. Sets of three 400 µm thick hippocampal slices from 7-weeks-old rats were pre-incubated for 4 hours in an interface chamber. After recording of baseline PSs every hippocampal slice experienced one of the following tetanization paradigms: early-LTP: single tetanization; late-LTP: triple tetanization or no tetanization (control). A) The diagram summarizes the increase of PS-amplitude after early-LTP induction paradigm. B) This diagram indicates the PS-potentiation in response to the late-LTP induction protocol. Thirty minutes after the first tetanization the slices were fixed and processed for Jacob and MAP2 immunolabeling as described in methods. C) A merged image of Jacob (green) and MAP2 (red) is presented at low magnification. The white box indicates an analyzed CA1 area. To the right, higher magnification confocal images for MAP2, Hoechst (blue) and Jacob as well as a merged image are shown. D) Representative overlay images of Jacob, MAP2 and Hoechst for non-, single and triple tetanized slices (control, early-LTP and late-LTP, respectively) are depicted. The averaged fluorescence intensity of nuclear Jacob staining was corrected against proximal part of basal dendrites (small square) as shown in the corresponding anti-Jacob confocal images. E) More than 20 nuclei from each slice were analyzed and the intensities averaged resulting in 12 single values for each group. In the bar diagram the fluorescence intensities are presented as percentage deviation from the average of the control group values. The number of analyzed nuclei is indicated in the graph. Mann Whitney test: **p<0.01: between control and L-LTP.

To further demonstrate the specificity and reliability of the nuclear translocation of Jacob in response to the induction of Schaffer-collateral-CA1 LTP we performed a series of experiments where we compared different LTP induction protocols leading to early- or late-LTP in interface slices of 7-weeks old rats. Moreover we applied a slightly different image analysis approach than the in Figure 1 by using a nuclear staining as a mask for the Jacob channel and thereby excluded the nuclear membrane from the analysis. In addition, we stained against MAP2 to indicate the dendritic and neuronal localization of Jacob (Fig. 3C). It is known that early-LTP does not require gene transcription, whereas late-LTP critically depends upon transcriptional regulation [13]. We found that the induction of early-LTP (Fig. 3A) had no significant effect on nuclear Jacob immunofluorescence in CA1 neurons (Fig. 3D–E). In contrast, triple tetanization that induced late-LTP (Fig. 3B) increased the nuclear Jacob immunolabel significantly (Fig. 3D–E). Thus, in two different slice preparations Jacob immunolabel is increased in the nucleus following induction of late-LTP, whereas a weaker tetanization protocol to induce early-LTP was not sufficient to drive Jacob into the nucleus.

### LTD induction does not drive Jacob into the nucleus

**Figure 4:**
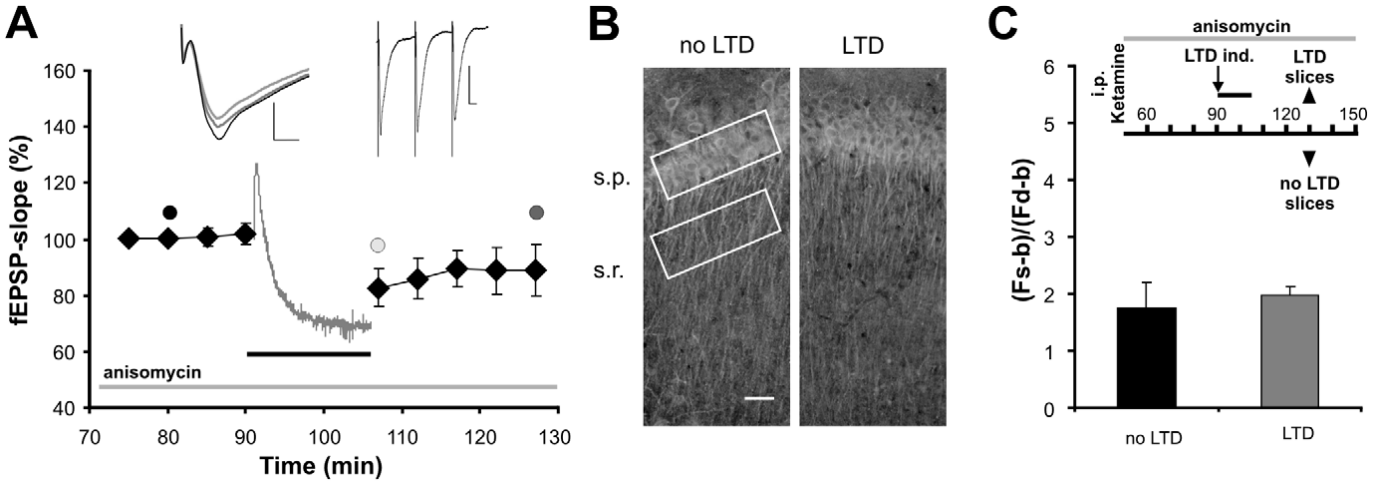
The Jacob distribution remains unaltered after LTD induction. A) Averaged normalized fEPSP-slope values (black filled diamonds) are shown. fEPSP-slope values during LTD induction (horizontal black bar) are depicted as gray dots. LTD was induced by 900 bursts and one burst consists of 3 stimuli at an inter-stimulus interval of 50 ms. The color of the fEPSP-transients in the left inlet corresponds to time points indicted by the respective black, light gray and dark gray filled circles. The second inlet shows fEPSPs of an initial burst during LTD induction. Slices for the immunohistological analysis of Jacob translocation were taken 40 minutes after the last baseline recording. B) Representative digital fluorescence images of Jacob immunofluorescence for the conditions “no LTD” and “LTD” are presented. The white boxes indicate ROIs taken for the measurement of averaged intensities within the s.p. and s.r. field. The white horizontal bar represents 10 µm. C) The bar diagram summarizes the immunofluorescence analyses of hippocampal slices without (black) and with LTD (dark gray) induction. The ratio of s.p. to s.r. was calculated after subtracting background values (b; minimum value in dendritic area) from the averaged fluorescence intensity of s.r. (Fd: dendritic fluorescence) and s.p. (Fs: somatic fluorescence). After LTD induction the ratio was not different from the ratio of “no LTD” slices. The inlet indicates the time for the various activities.

**Figure 5:**
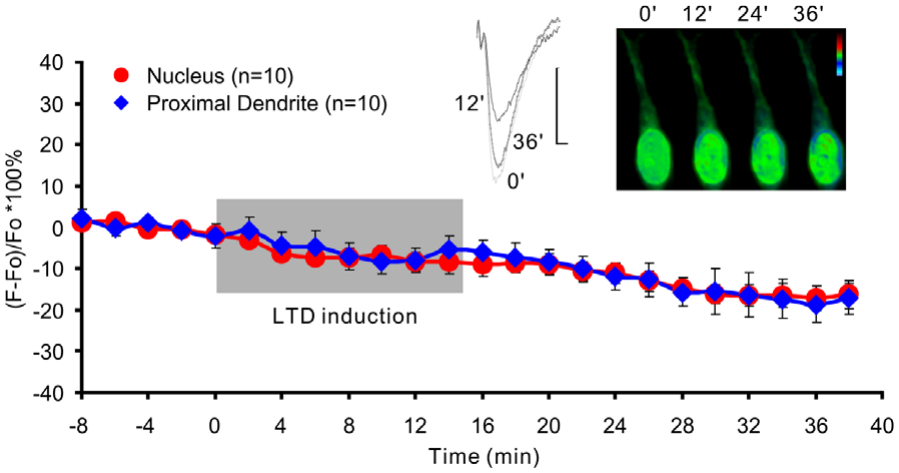
Low frequency induction of LTD does not induce Jacob-GFP translocation into the nucleus. The time course of normalized fluorescence intensities is presented for the nucleus (red filled circle) and the proximal dendrite (dark blue diamond). The recordings were acquired in the presence of anisomycin (Anis.: 50 µM). The gray box indicates the time of LTD induction (900 bursts at 1 Hz, one burst consists of 3 stimuli at an interstimulus interval of 50 ms). Representative fEPSP-transients for indicated time points are shown. Vertical and horizontal scale bars indicate 0.5 mV and 2 ms, respectively. The other inlet depicts representative F/F_o_*100% confocal images of a neuron expressing Jacob-eGFP before and after LTD induction. The vertical colored bar indicates green as 0, and dark blue as −20% and red as +20%. The image analysis was done as indicated in Figure 2.

To address whether the nuclear translocation of Jacob in hippocampal slices is specifically related to NMDA-receptor dependent LTP induction we also evoked NMDA-receptor dependent LTD (**Fig. 4A**). Immunohistochemical analysis showed that in these experiments the ratio of Jacob-immunofluorescence in the str. pyramidale as compared to the str. radiatum did not change (**Fig. 4B–C**), thus indicating that no detectable translocation after LTD induction takes place. In addition, over-expressed Jacob-GFP did not accumulate in the nucleus after induction of LTD, which did not evoke a decrease of fluorescence signal intensity in proximal dendrites of str. radiatum in time-lapse imaging experiments (**Fig. 5**).

**Figure 6:**
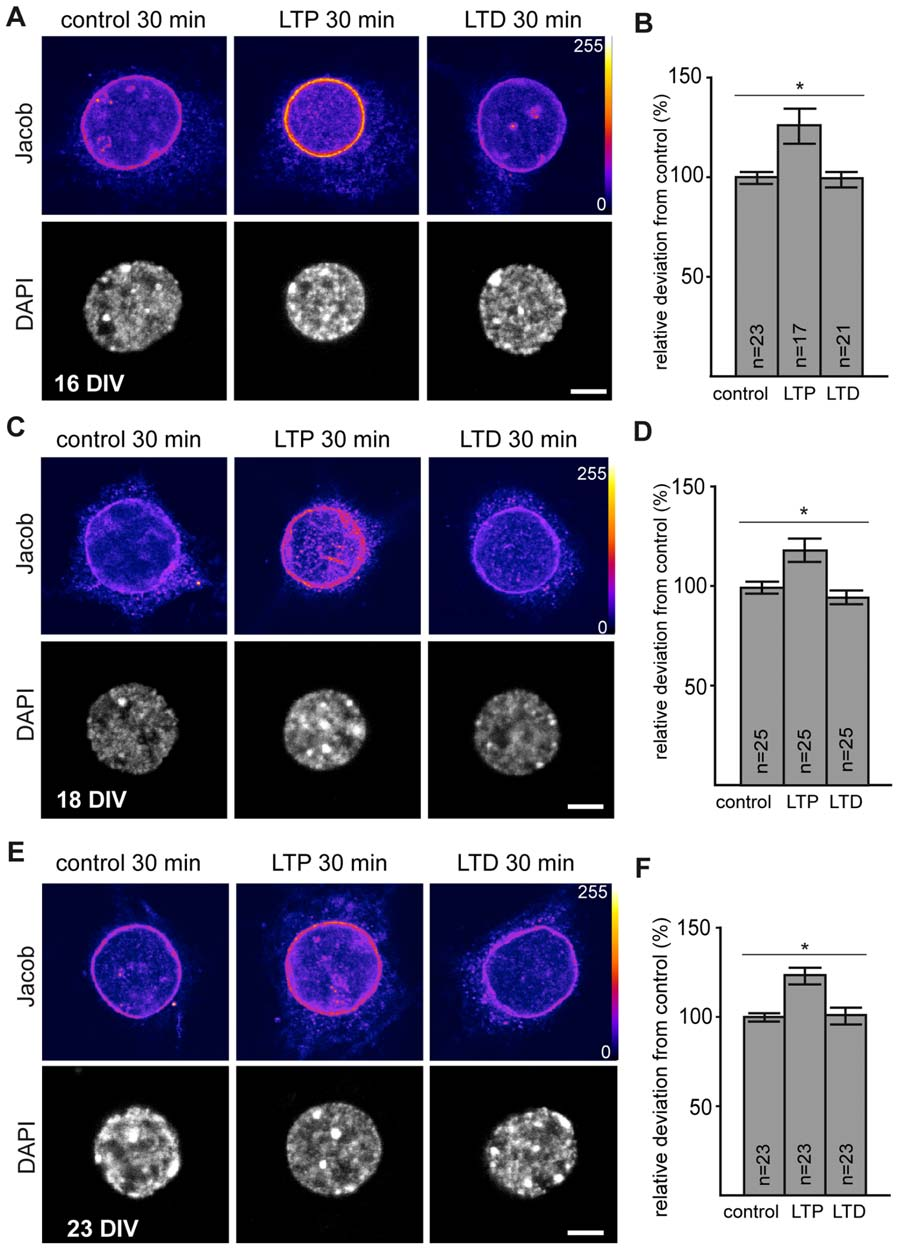
Jacob translocates to the nucleus after induction of chemical LTP but not chemical LTD in adult primary hippocampal neurons. 10 minutes prior to stimulation the Neurobasal medium was replaced by magnesium free buffer solution containing TTX, strychnine and bicuculline. 200 µM glycine or co-application of 20 µM glycine and 20 µM NMDA were applied to modulate synaptic transmission; known to induce chemical LTP or chemical LTD, respectively. Five minutes after stimulation the conditioning medium was replaced and cells further incubated for additional 25 minutes and then fixed. A,C,E) Representative anti-Jacob and DAPI fluorescence images of 16, 18 and 23 DIV cultures for control, chemical LTP and chemical LTD, respectively, are shown. B, D, F) The diagrams summarize the relative deviation of nuclear Jacob fluorescence under different experimental conditions. Independent from the age of culture, chemical LTP induction induced a significant accumulation of Jacob, but induction of chemical LTD did not change Jacob immunosignal under all tested conditions. Statistical significance was determined by ANOVA with Bonferroni post-hoc t-test. *p<0.05. Scale bar is 5 µm.

These results were interesting because previous work has shown that another putative synapto-nuclear messenger, CREB2, exclusively transits to the nucleus in hippocampal primary neurons after chemical LTD but not chemical LTP [8]. To complement the findings from hippocampal slices we investigated whether chemical induction of LTP and LTD using the protocol developed by Lu et al. [21] that was also employed in the CREB2 study [8], will lead to similar results. We indeed found that bath application of 200 µM glycine, which has been shown to induce chemical LTP [21], induced a prominent nuclear accumulation of Jacob 30 minutes following stimulation in hippocampal primary neurons at day 23 in vitro (days in vitro: DIV) (**Fig. 6E–F**). In contrast, bath application of 20 µM glycine/20 µM NMDA, a protocol that was shown to induce chemical LTD [21], was not followed by a nuclear accumulation of Jacob, as previously reported for CREB2 (**Fig. 6E–F**).

### Nuclear accumulation of Jacob depends upon NMDA and glycine concentrations as well as the age, handling and prior activity of cultures

**Figure 7:**
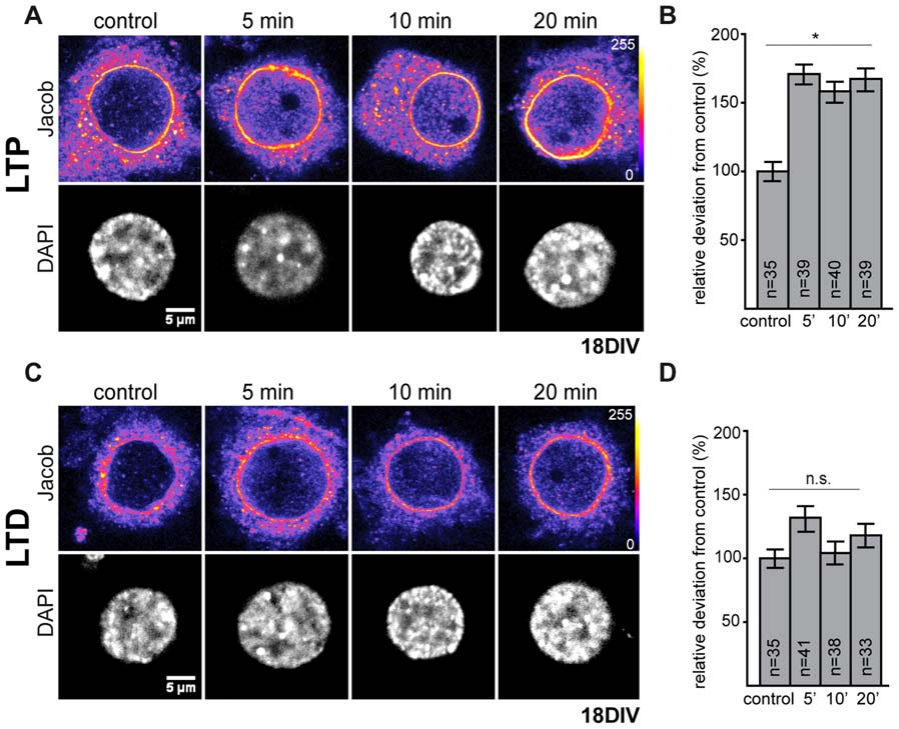
In DIV 18 cultures Jacob rapidly accumulates in the nucleus after chemical induction of LTP but not LTD. A) Chemical LTP was induced by application of 200 µM glycine, followed by fixation at indicated time points. B) The increase in nuclear Jacob immunoreactivity became significantly different from controls already 5 minutes after chemical LTP induction. C) Chemical LTD was induced through application of 20 µM glycine and 20 µM NMDA followed by fixation at the indicated time points. D) The diagram summarizes the normalized increase in Jacob immunosignal. Five minutes after stimulation a non-significant increase of nuclear Jacob has been observed. Bars represent mean± s.e.m. Statistical significance was analyzed by ANOVA with Bonferroni post-hoc t-test. *p<0.05. Scale bar is 5 µm.

A major concern in studies using cultured primary neurons is that differences in culture conditions, handling procedures and stimulation paradigms will cover the real dynamics of nucleocytoplasmic shuttling in response to synaptic activity. We therefore performed a series of further control experiments to learn more about the conditions that elicit a redistribution of Jacob towards the nucleus. NMDA-receptor expression is developmentally regulated with a change in the ratio of NR2A/NR2B subunits and their synaptic and extrasynaptic localization [22]–[25]. The age of the culture might therefore have an important influence on the dynamics of Jacob's nuclear accumulation. To exclude this possibility we repeated the experiments in cultures at DIV 16 and 18 (**Fig. 6A–D**) with essentially the same result that was observed with cultures at DIV 23 (**Fig. 6E–F**). No increase in nuclear Jacob immunofluorescence was found in cultures stimulated with the LTD protocol, whereas neurons treated with the LTP protocol exhibited a prominent accumulation of Jacob in the nucleus (**Fig. 6A–F**). Interestingly, when we followed up the dynamics of the translocation, we found a statistically significant increase of nuclear Jacob immunofluorescence already 5 minutes after LTP stimulation in cultures at DIV 18 (**Fig. 7A–B**), while no significant increase was found with the LTD protocol at any time point investigated (**Fig. 7C–D**).

**Figure 8:**
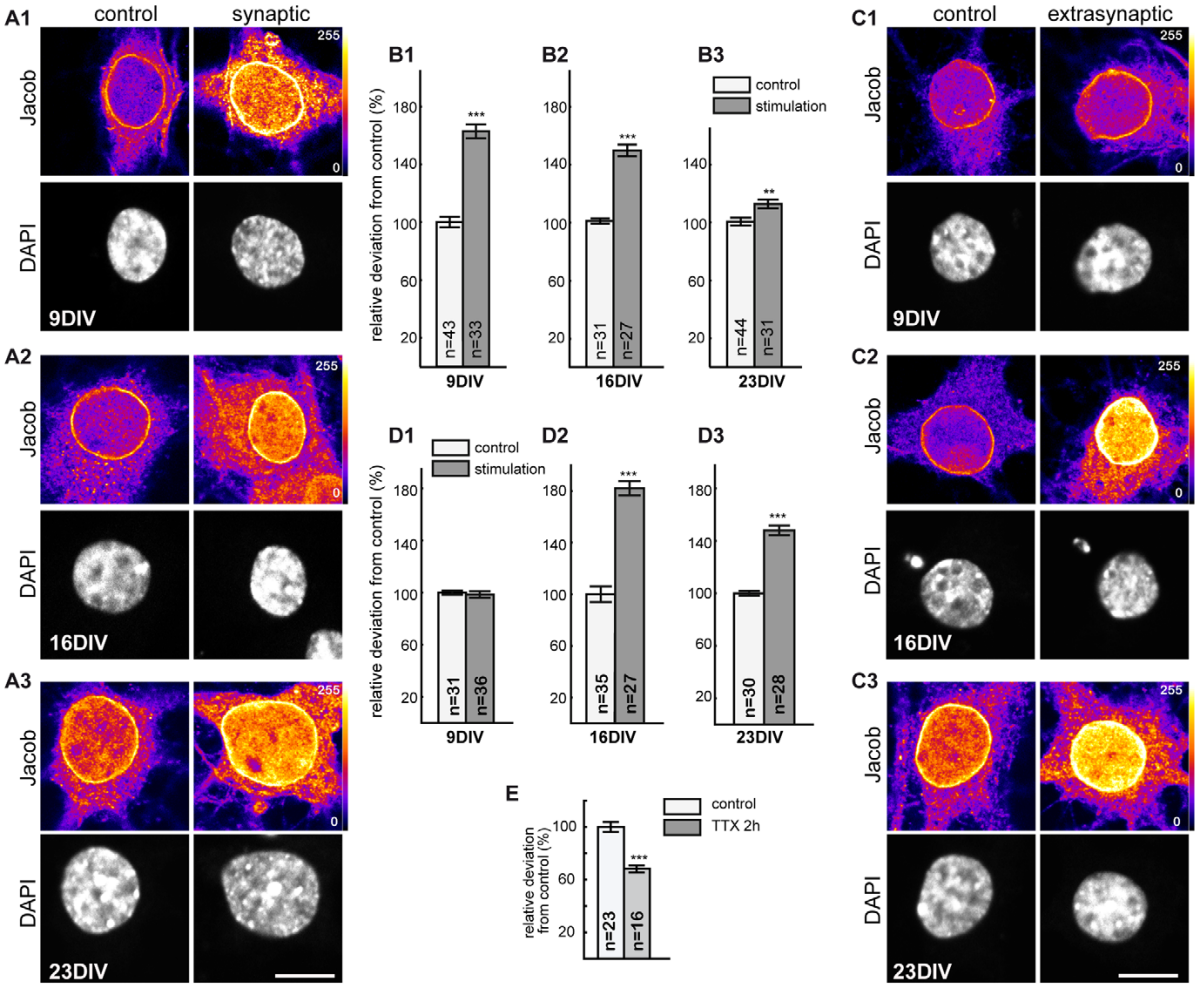
Nuclear Jacob immunoreactivity at different culture ages following synaptic or extrasynaptic NMDA receptor activation. A1–A3 and C1–C3) Representative fluorescence images for anti-Jacob and DAPI stained hippocampal primary neurons at 9, 16 and 23 DIV. Cultures were stimulated with protocols activating synaptic (bicuculline, 4-AP) and extrasynaptic NMDA receptors (bicuculline, 4-AP, MK801; followed by 100 µM NMDA). Note the age dependent increase in Jacob nuclear immunoreactivity at basal conditions. Synaptic stimulation induced a Jacob translocation in cultures at DIV 9 (A1 and B1) but not after extrasynaptic stimulation (C1 and D1). B1–B3) The efficiency of synaptic stimulation on nuclear Jacob immunosignals decreased with culture age. D1–D3) At DIV 23 extrasynaptic NMDA receptor stimulation increased nuclear Jacob signal. E) TTX treatment for 2 hrs significantly reduced nuclear Jacob. **p<0.01 ***p<0.001. Scale bars are 10 µm.

Nucleocytoplasmic shuttling of Jacob depends upon activation of synaptic and more prominently of extrasynaptic NMDA-receptors [7]. The induction of LTP/LTD at the Schaffer-collateral input to CA1 neurons appears to be dependent on activation of synaptic NMDA-receptors [11], [26], [27], although extrasynaptic NMDA receptors might also be activated to a variable degree [27], [28] but this is most likely only important for the magnitude but not maintenance of LTD [27]. In contrast, albeit chemical induction of LTP with 200 µM glycine is thought to trigger Ca^2+^-influx through synaptic NMDA-receptors, bath application of 20 µM glycine/20 µM NMDA might also induce LTD through activation of peri - or extrasynaptic NMDA-receptors [21]. We therefore wondered whether differences in stimulation protocols and/or maturation of the culture might be responsible for the discrepant results regarding the nuclear trafficking of Jacob after activation of extrasynaptic NMDA receptors in the present and our previous reports. Our previous experiments were done with neurons in cultures at DIV 14–16 using bath application of higher NMDA concentrations or selective activation of synaptic/extrasynaptic NMDA-receptors [7], [9], [16]. Using similar protocols, we found that at DIV 16 extrasynaptic (**Fig. 8C2–D2**), and to a lesser degree synaptic NMDA-receptor activation (**Fig. 8A2–B2**), induces the nuclear translocation of Jacob. However, at DIV 9 activation of synaptic NMDA-receptors leads to nuclear import of Jacob (**Fig. 8A1–B1**) but not extrasynaptic stimulation (**Fig. 8C1–D1**), whereas at DIV 23 we obtained the opposite result (**Fig. 8A3–B3,C3–D3**). Thus, extrasynaptic NMDA-receptor activation is efficient to drive Jacob into the nucleus in mature cultures at DIV23 when the chemical LTD experiments were done (**Fig. 8**). The minor, albeit significant increase of nuclear Jacob immunofluorescence after activating synaptic NMDA-receptors is at this age of the culture probably due to the higher basal synaptic activity, which is followed by higher nuclear levels of Jacob under control conditions (Karpova, Mikhaylova, Kreutz, unpublished observations). The buffers for induction of LTP contain the sodium channel blocker TTX and we indeed found that nuclear immunofluorescence levels of Jacob are significantly reduced when we blocked action potentials for 2 hours with TTX treatment (**Fig. 8E**).

**Figure 9:**
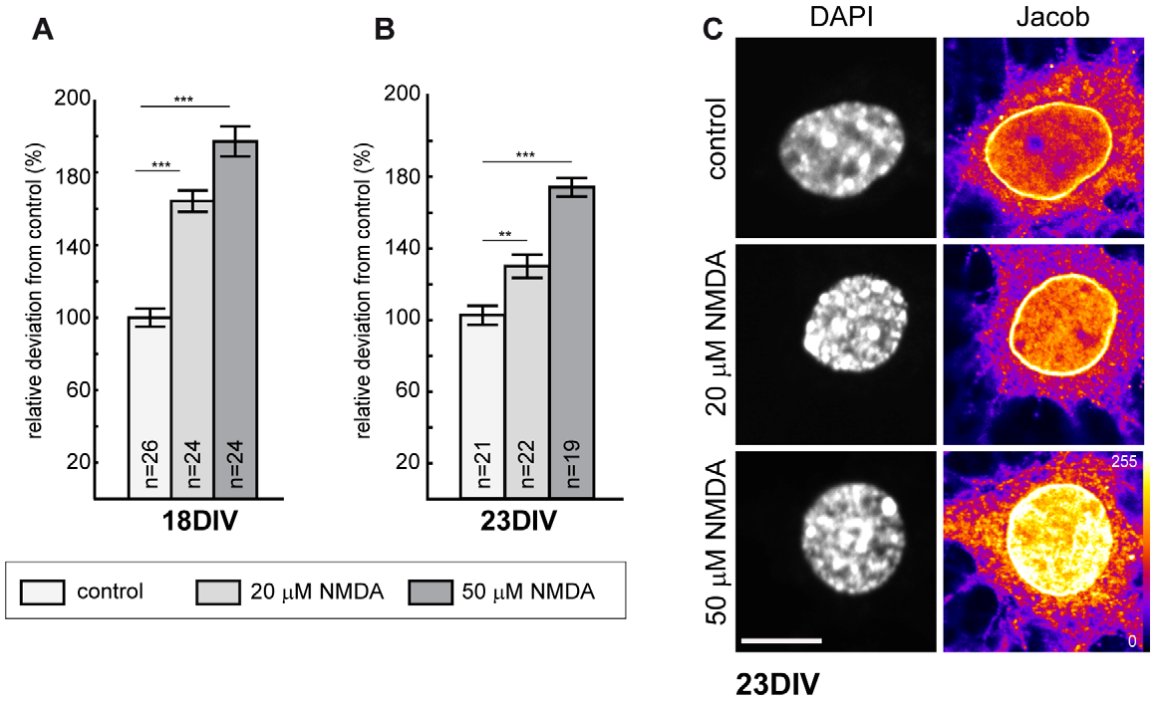
NMDA dose-dependent increase in Jacob nuclear immunoreactivity of primary hippocampal neurons. Hippocampal primary neurons were maintained in 1 ml of neurobasal medium (NB). 12–16 hours prior stimulation the volume of NB medium was increased to 1.6 ml. Then 800 µl of conditioned NB medium was removed and NMDA applied. Five minutes later the NMDA-NB medium was replaced with the 800 µl conditioned NB medium and 25 minutes later the neurons were fixed. A) The diagram summarizes normalized nuclear Jacob immunofluorescence at DIV 18 after bath application of 20 µM or 50 µM NMDA. B) The bar diagram indicates the gain in the nuclear fluorescence signal after treatment with 20 µM or 50 µM NMDA in comparison to drug free conditions. Note the increased nuclear accumulation of Jacob at DIV 18. C) Representative images of Jacob nuclear immunoreactivity and DAPI staining in hippocampal primary neurons at DIV 23 under control, 20 µM and 50 µM NMDA conditions, respectively. ***p<0.001, **p<0.01. Scale bar is 10 µm.

The stimulation protocol for extrasynaptic NMDA receptors involves bath application of 100 µM NMDA for 3 minutes. We therefore wondered whether lower concentrations of NMDA like those used for induction of chemical LTD are effective. Our previous experiments using bath application of NMDA were performed with Neurobasal medium, which contains high concentrations of glycine (400 µM). We indeed found that bath application of 20 and 50 µM of NMDA in Neurobasal medium for 5 minutes induced a prominent accumulation of Jacob in the nucleus of DIV 18 and DIV 23 hippocampal primary neurons (**Fig. 9A–C**).

## Discussion

The present study in conjunction with previously published work [8] suggests that different putative synapto-nuclear protein messenger translocate to the nucleus in response to certain types of activity-dependent synaptic plasticity. Collectively these studies suggest that synapto-nuclear protein messenger have the potential to induce specific nuclear responses to different types of synaptic input and it is likely that this correlates with the induction of the different type of gene expression observed in LTP and LTD. Most studies so far were performed in neuronal primary cultures and used stimulation paradigms of limited physiological significance. In this study, we therefore fill an important gap regarding the proposed functional role of synapto-nuclear protein messengers in cellular plasticity. We can show that at least one member of this growing group exhibits highly dynamic trafficking from dendrites to the nucleus already during the tetanization period of LTP in hippocampal slices *in vitro*.

Importantly, nuclear trafficking of Jacob is already detected during the induction of LTP. Frey and co-workers have shown that the requirement for transcription in order to maintain LTP may have a critical time window [17], [18] because the blocker of gene transcription actinomycin D was only effective in influencing the maintenance of LTP when applied before tetanization, while it was ineffective when it was administered shortly after tetanization [18]. Accordingly, one might argue that trafficking of synapto-nuclear protein messengers might be to slow to enter the nucleus to be involved in plasticity-related gene expression in this cellular model of learning and memory. Our results strongly suggest that this is not the case. We could show that Jacob deriving from dendrites is capable of entering the nucleus within a few minutes during tetanization of the slices. Interestingly, during the course of these experiments, we realized that already the preparation of slices led to higher levels of Jacob in the nucleus than seen in sections stained with the same Jacob antibody from perfused animals (data not shown). Thus, the increase in nuclear Jacob might be even larger in models of *in vivo* LTP or LTD where no uncontrolled release of glutamate due to neuronal injury during preparation of the slices will drive Jacob into the nucleus. It should also be noted that Jacob immunostainings suggest a prominent association of the protein with the nuclear membrane. It will be interesting to analyze whether the protein is mainly associated with the inner or the outer nuclear membrane.

Taken together, these findings suggest that Jacob might be involved in the control of gene expression to maintain LTP type of synaptic plasticity. It will be interesting to investigate whether other putative synapto-nuclear messenger than CREB2 or Jacob exhibit similar specificity concerning activity-dependent nuclear import following induction of synaptic plasticity. Since trafficking from distal dendrites supposedly requires association with specific importins, the question arises at which levels these different forms of plasticity regulate these processes. One possibility could be that different importins are involved in synapse-to-nucleus trafficking during induction of LTD and LTP. All members of the importin-α and -β family are expressed in brain at different levels [29] and previous work has shown that induction of chemical LTP in hippocampal slices leads to increased nuclear import of importin-α1, α2 and β1 [14]. In addition, NMDA receptor-dependent nuclear import of importin-α1 and -α2 as well as importin-β1 has been shown in hippocampal primary neurons [7], [14]. CREB2 has been shown to specifically associate with importin-α1 and -α6 but not -α2, -α3 and -α4 [8]. Jacob associates with importin-α1, however, it is unclear whether it will bind other importins as well. Hence, nuclear NF-κB accumulation appears to primarily require binding to importin-α3 and -α4 [30] but other family members might contribute here as well. Moreover, at present it is also unclear whether importins and these putative synapto-nuclear protein messengers are exclusively associated with excitatory synapses that are thought to be the subject of LTP and LTD. Interestingly however a recent report documents that importin-α1 directly associates with the cytoplasmic tail of a splice isoform of the NR1 subunit of NMDA-receptors [15] providing a direct link to NMDA-receptor signaling. It was also reported that importin-α1 dissociate from the NMDA-receptor in an activity and PKC-dependent manner. Interestingly, in accord to the observations made in the present study no dissociation of importin-α1 from the NR1 subunit was found with stimulation protocols inducing gene transcription independent early-LTP whereas a dissociation was seen after induction of late-LTP [15]. It is, however, unclear whether all importins associate with the same binding motif and how LTP and LTP inducing NMDA-receptor signals could provide specificity for the dissociation of certain importins. The NMDA-receptor induced Ca^2+^ signals that bring about either LTP or LTD differ only in their timing and duration. LTP is triggered by Ca^2+^ signals on the micromolar scale for shorter durations, whereas LTD is triggered by changes in free Ca^2+^ concentrations on the nanomolar scale for longer durations [31]. It is therefore conceivable that the regulation of synapto-nuclear trafficking happens at the level of posttranslational modifications of importins or the messenger itself.

The surprising result that Jacob translocates rapidly to the nucleus after induction of late-LTP but not LTD also raises some interesting questions about the functional role of the protein. Our previous studies suggested that Jacob is a messenger of cell death after activation of extrasynaptic NMDA-receptors and might be involved in the neurodegenerative CREB-shut off pathway [7], [16]. However, we also observed a less prominent nuclear accumulation of the protein after triggering the activity of synaptic NMDA-receptors [7]. In this study we could confirm both findings and it will be interesting to elucidate whether nuclear Jacob regulates different types of gene expression depending upon the synaptic or extrasynaptic location of the activated NMDARs. The different characteristics of both signals might translate into different posttranslational modifications that could help to distinguish the synaptic or extrasynaptic NMDARs. It appears that the extent of Jacob's nuclear accumulation after triggering synaptic and extrasynaptic NMDA-receptors depends on the age and the prior activity of the culture as well as the concentration of NMDA and glycine in the extracellular solution. Interestingly, extrasynaptic NMDA-receptor activation was inefficient to induce nuclear import of Jacob at DIV 9. At this stage the CREB shut-off pathway is not yet functional [32], [33] and in addition Jacob protein levels are low (Karpova, Mikhaylova, Kreutz, unpublished observations). Future studies might clarify whether both phenomena are causally related. However, the reason why NMDA-receptor dependent LTD neither in slices nor in primary cultures is incapable to induce Jacob's nuclear import is at present still unclear and warrants further investigation.

## Materials and Methods

### Ethics Statement

In the present experiments, animal care and procedures were approved and conducted under established standards of the Institutes of Brain Science and State Key Laboratory of Medical Neurobiology of Fudan University, Shanghai, China and of the German federal state of Sachsen-Anhalt, Germany in accordance with the European Communities Council Directive (86/609/EEC).

### Electrophysiological methods

Hippocampal LTP was investigated in acutely prepared hippocampal slices from 2-3 weeks old male Wistar rats (CAS, P.R. China). Hippocampal slices were prepared as described previously [34], [35]. Briefly, after anaesthetization using ether, the rat was decapitated and the brain was removed quickly and immersed in carbogenated ice-cold Gey's solution (composition in mM: 130 NaCl, 4.9 KCl, 1.5 CaCl_2_·2H_2_O, 0.3 MgSO_4_·7H_2_O, 11 MgCl_2_·6H_2_O, 0.23 KH_2_PO_4_, 0.8 Na_2_HPO_4_, 5 glucose, 25 HEPES, 22 NaHCO_3_, pH 7.32). After dissection of the cerebellum a part of the entorhinal cortex was cut off and the hemispheres were glued with the cross section on the slicing platform of the sectioning system (Vibratome 3000, St. Louis, MO, USA). Transverse hippocampal slices (350 µm) were transferred to a custom made interface type recording chamber and incubated for at least 2 hours at 32 degree under constant perfusion with carbogenated artificial cerebrospinal fluid (ACSF containing in mM: 110 NaCl, 5 KCl, 2.5 CaCl_2_·2H_2_O, 1.5 MgSO_4_·7H_2_O, 1.24 KH_2_PO_4_, 10 glucose, 27.4 NaHCO_3_, pH 7.3).

In time-lapse imaging experiments field excitatory postsynaptic potentials (fEPSPs) were evoked by stimulation of Schaffer-collateral fibers with biphasic rectangular current pulses (100 µs/polarity) in a range of 15–30 µA through glass pipettes with a resistance of 0.5 MOhm. Field EPSPs were recorded via glass pipettes filled with ACSF solution from the str. radiatum. using an AxonClamp 200B amplifier (Axon, US).

In experiments looking for the translocation of endogenous Jacob the fEPSPs were evoked and recorded using A-M systems stainless steel electrodes (A-M Systems, Sequim, WA, USA, 0.5 MOhm and 1 MOhm, respectively), EXT-20F amplifier (npi electronic GmbH, Tamm Germany) and the stimulus isolator MT8000 (Multichannel, Germany). Stimulation strength was adjusted to 40% of the maximum fEPSP-slope values. Responses to test stimuli were measured every minute or 5 minutes throughout experiments. Late-LTP was induced by a strong tetanization consisting of three 1-s trains at 100 Hz every 10 minutes. Late-LTD was induced by 900 bursts at 1 Hz (one burst consists of 3 stimuli at 50 ms inter-stimulation intervals). This LTD induction protocol induced a long-lasting LTD in vitro that was described to last over 8 hours [36].

The averaged fEPSP-slope values were depicted in diagrams as mean±s.e.m. The recorded field potentials were digitized at a sample frequency of 10 kHz by Digidata 1400plus AD/DA converter (Axon, USA) or CED 1401plus (Cambridge Electronics Design, Cambridge, UK).

In order to compare Jacob immuno-signals after induction of early- and late-LTP with non-tetanized controls (Fig. 2) a set of three hippocampal slices were prepared from 7-weeks-old Wistar rats. The slices were incubated in an interface chamber for at least 4 hours at 32°C. The ACSF contained (in mM): 124 NaCl, 4.9 KCl, 1.2 KH_2_PO_4_, 2.0 MgSO_4_, 2.0 CaCl_2_, 24.6 NaHCO_3_, 10 D-glucose. The carbogen consumption was 30–35 l/h and the ACSF flow rate was 0.6–0.7 ml/min. Stainless-steel electrodes (A-M Systems, Sequim, WA, USA) were positioned in all slices of the set within the str. radiatum for stimulation and in the str. pyramidale for population spike (PS) recording. Evoked field potentials were amplified by a custom-made amplifier (IfN, Magdeburg, Germany), digitized with a CED 1401 A/D converter and acquired and analyzed using custom-made software (PWIN, IfN, Magdeburg, Germany). Following preincubation, the test stimulation strength was determined to elicit a PS of 40% of its maximal amplitude for control recordings and 25% of the maximal PS for LTP-experiments. Late-LTP was induced by three 1-s trains at 100 Hz (“strong” tetanus (STET), impulse duration: 0.2 ms/polarity; intertrain interval: 10 min). In experiments with induction of early-LTP, a weak tetanization (WTET) protocol consisting of one 100 Hz train (21 biphasic constant-current pulses; pulse width duration 0.2 ms/polarity) was employed. At the time points 1, 3, 5, 11, 15, 21, 25, 30 minutes field potentials were recorded four times at 0.2 Hz (impulse duration: 0.1 ms per polarity) and averaged. Thirty minutes after first tetanization slices were fixed in 4% PFA and proceeded for cryosectioning, immunostaining and confocal imaging.

### Immunocytochemistry of hippocampal slices

40 minutes after last baseline recording of fEPSP recordings, hippocampal slices were fixed for 30 minutes in a 2.5% paraformaldehyde, 0.1 M phosphate buffered saline (PBS, pH 7.4) at ambient temperature, transferred to PBS with 30% sucrose and then stored at 4°C. Forty-five micrometer sections were prepared using a cryostat (CM 1900, Leica, Germany). The surface sections were separated from the sections of the inner part of the hippocampal slices where the tip of the electrode was placed. The sections were placed for 1 h at room temperature in PBS containing 2% glycine, 0.2% gelatine, 50 mM NH_4_Cl, 2% BSA and 0.3% Triton X-100. The primary antibodies (polyclonal rabbit anti-Jacob ([7] Jb150, 1∶100) were diluted in the blocking solution and slices were incubated over night at 4°C. The sections were rinsed three times with PBS before 4 hours of incubation with Alexa-dye conjugated secondary antibodies (anti-rabbit IgG488 from Molecular Probes, USA, dilution 1∶200) in the blocking buffer at room temperature. In case of nucleus counterstaining DAPI was added to PBS and the slices kept in this solution for 5 minutes. Three washing steps were performed before the stained sections were mounted with prolong antifade (Molecular Probes, USA) on cover slips.

Fluorescence images of the slices were acquired using Olympus laser-scanning microscope and/or video imaging system (Olympus: DP71 CCD camera) mounted on BX51 Upright microscopes. The excitation level and the acquisition were kept constant for all samples. 8 bit 4080×3072 digital pictures were acquired using a 20fold objective. Region of interests (ROIs) were placed along the str. pyramidale (s.p.) and str. radiatum (s.r.). For normalization purposes a ratio of s.p. to s.r. was calculated after subtracting background values (b; minimum value in dendritic area) from the averaged fluorescence intensity of s.r. (Fd: dendritic fluorescence) and s.p. (Fs: somatic fluorescence). The ratio (Fs-b)/(Fd-b) indicates an increase or decrease of fluorescence intensity in relation to each other.

Hippocampal slices after induction of early- or late-LTP and their control were immunolabeled against Jacob and MAP2, and stained with Hoechst. The str. pyramidale of the CA1 region was scanned with a 4× optical zoom using a 63× oil immersion objective. Only the middle approximately 1 µm thick area of cell nuclei (maximum intensity projection of three Z-sections) was used for analysis to avoid detection of Jacob immunofluorescence from nuclear membranes. Nuclear staining by Hoechst was used as a mask to identify the inner part of nucleus and to exclude the nuclear rim. For each individual section the mean fluorescence intensity of nuclear Jacob staining was normalized against the mean intensity of basal dendrites (small square in Figure 3). To minimize staining and fluorescence variation sets of slices were handled within the same day. The fluorescence intensities of more than 20 nuclei from each slice were determined and averaged and taken as one experiment.

### Image acquisition and analysis of time-lapse recordings of Jacob translocation

The time course of fluorescence changes in wild-type Jacob-GFP expressing CA1 neurons was acquired by scanning of 10 planes using confocal microscope every 2 min. The distance between the first and the last optical plane was about 70 µm. Fluorescence intensity values (averages over regions of interest, ROIs, as indicated) were calculated for every time point after imaging processing: 1) averaging of 2 to 3 planes that encompasses a nucleus, 2) crop of a nucleus and proximal dendrite, 3) alignment of averaged planes along the time, 4) measuring of fluorescence intensity of ROIs located on the nucleus (excluding nuclear membrane), proximal dendrites and background. Fluorescence intensity values are expressed as percentage changes from baseline and normalized against background intensity values. Image processing and analysis was conducted using open source imaging software ImageJ3.5.

### Generation of the reporter construct, preparation of Semliki Forest particles and *in vivo* transfection of CA1 neurons

The coding sequences of WT-Jacob-eGFP and eGFP were amplified by PCR and cloned separately into the SmaI site of the Semliki Forest Vector pSFV1 (Clontech Invitrogen). Semliki Forest particles were prepared as described in Ehrengruber et al. [37] using the pSFV-helper2 for structural proteins. After in-vitro transcription both the RNAs of pSFV-wild-Jacob-GFP and pSFV-Helper 2 were co-transfected into HEK-293T cells with Lipofectamine 2000 (Invitrogen) according to the supplier manual. After 36 hours, the culture medium containing the budded particles was harvested and passed through 0.22 µm filters. For long-term storage at -80 degree DMSO was added to get 7 vol/vol percentages. Thirty minutes before conducting *in viv*o injection, the particles were activated by alpha-chymotrypsin (Sigma, US; CAS No. 9004-07-3; 500 mg/L) for 30 minutes at room temperature and the reaction inactivated by aprotinin (Sigma, US; CAS No 9087-70-1; 250 mg/L).

For the purpose of intrahippocampal in vivo injection Wistar rats (2–3 weeks old) were anesthetized with ketamine (70 mg/kg) by intraperitoneal injection and secured in a stereotaxic frame. The injection coordinates were determined using the point of intersection of the sagittal suture with the best fit along the coronal suture (Bregma) as a reference for the lateral and anterior-posterior coordinates (AP: −5.4 mm; L: ±5.2 mm). The dura mater was carefully punctuated to expose the brain surface, which was used as a reference point for the vertical coordinates (V: −4.0 mm). For intrahippocampal injection glass pipettes with a tip diameter of about 30 µm were filled with activated particle solution and inserted into the hippocampus through a 0.5 mm diameter hole in the skull. The particle solution was then injected three times for 5 minutes at a rate of about 0.5 µl per minute. The injection pipette was left at the final position for about 20 minutes.

### Primary cell culture, chemical LTP/LTD induction, NMDA stimulation, synaptic versus extrasynaptic stimulation and quantitative immunocytochemistry

Hippocampal primary cultures were prepared according to Goslin and Banker [38] using embryonic day 19 old rat embryos. Cells were plated in a density of 40.000 cells per 18 mm coverslip, grown in 1 ml of neurobasal medium (NB, Gibco) supplemented with B27 medium. Ara-C (cytosine β-D-arabinofuranoside) at a final concentration of 5 µM was included in the culture medium at DIV 7 to suppress glia proliferation. Anisomycin at a final concentration of 7.5 µM was applied to the cultures to suppress de novo protein synthesis immediately before onset of the experiment. Chemical LTP or LTD was induced following a protocol from Lu et al, [21]. Hippocampal neurons (DIV 16, 18, 23) were incubated in a pre-warmed extracellular solution (in mM: NaCl 140, CaCl_2_ 1.3, KCl, 5.0, HEPES 25, glucose 33, TTX 0.0005, strychnine 0.001, bicuculline methiodide 0.02; pH 7.4, osmolarity 325–335 mosmol^−1^) for 10 min prior to application of 200 µM glycine for 3 min (LTP) or 20 µM NMDA and 20 µM glycine for 3 min (LTD). The stimulation solution was removed and replaced by pre-warmed extracellular solution at the time points indicated (5, 10, 20, 30 min).

Bath NMDA stimulation was done as described before [7]. Briefly, one day prior to stimulation the volume of the cultured media was adjusted to 1.6 ml with fresh neurobasal medium. In order to have the same buffer conditions during and after the stimulation 800 µl of conditioned NB media was removed and used thereafter for NMDA bath application. Hippocampal neurons (DIV 18, 23) were treated with 20 µM and 50 µM NMDA (Sigma, Germany) for 5 min, washed and subsequently incubated in conditioned NB media for additional 25 minutes.

The selective activation of synaptic and extrasynaptic NMDA receptors followed published procedures [7], [39]. To activate synaptic NMDA receptors the hippocampal cultures were treated with 50 µM bicuculline (Tocris) with supplementation of the weak potassium-channel blocker 4-aminopyridine (4-AP, 2.5 mM, Sigma-Aldrich). Substances were added directly into the culture medium and cells were fixed 30 min after the treatment. For extrasynaptic NMDA receptors activation 9 DIV, 16 DIV and 23 DIV hippocampal neurons were sequentially exposed to 50 µM bicuculline, 2.5 mM 4-AP and 10 µM MK-801 to irreversibly block activated synaptic NMDA receptors. Neurons were then incubated for 30 minutes, washed by conditioned neurobasal medium and then 100 µM NMDA for 3 minutes was applied to stimulate the extrasynaptic population of NMDA receptors. After bath application of NMDA, culture medium was exchanged and neurons were incubated for additional 25 minutes before fixation.

Cells were fixed with 4% paraformaldehyde (PFA) for 10 min, washed 3 times with phosphate buffered saline (PBS), permeabilized with 0,25% TritonX-100 in PBS for 10 min, washed again and blocked for 1 hour in blocking buffer (2% glycine, 2% BSA, 0,2% gelatine, 50 mM NH_3_Cl; pH 7,4). Primary antibody were diluted in the blocking buffer (Jb150 1∶300) and incubated overnight at 4°C. After extensive wash the secondary antibodies (in blocking buffer) were applied for 1.5 hours at RT in darkness. Coverslips were washed again 3 times with PBS for 10 min and 1 time with water and mounted on the slides with Vectashield® Mounting Medium with DAPI (Vector Laboratories, Burlingame, USA).

Images were taken using a Leica DMRXE microscope equipped with a Blue-Violet laser (405 nm), Krypton-Argon-Ion laser (488/568/647 nm) and an acousto-optic tunable filter for selection and intensity adaptation of laser lines. The nuclei were identified utilizing 4′,6-diamidino-2-phenylindole (DAPI) staining. The images were analyzed with ImageJ software (NIH, Bethesda, USA). Identified nuclei were set as a region of interest (ROI) and applied as mask to the Jacob staining. Nuclear Jacob IR levels were determined by calculating the mean pixel intensity (average z-projection) from three nuclear optical sections. The differences between groups are described as percentage deviations from the average of the non-treated control.

### Statistical analysis

Data acquisition and analyses were carried out using pCLAMP and pFIT (Axon, US) or “PWIN” software (IfN, Magdeburg, Germany) and SPSS that allow for the measurement of the initial fEPSP-slope or PS-amplitude and the calculation of descriptive statistics. Data were normalized to baseline values and expressed as percentage as mean±s.e.m deviation. Comparisons of different time points and/or different groups were done by using either Mann-Whitney U-test or ANOVA and subsequent Bonferroni post-hoc T-tests. P values of ≤0.05 were considered statistically significant.
