# Correction: Nuclear Translocation of Jacob in Hippocampal Neurons after Stimuli Inducing Long-Term Potentiation but Not Long-Term Depression

**DOI:** 10.1371/annotation/8d9dd14e-08f6-4f7e-b881-ba2839724e23

**Published:** 2011-03-01

**Authors:** Thomas Behnisch, PingAn YuanXiang, Philipp Bethge, Suhel Parvez, Ying Chen, Jin Yu, Anna Karpova, Julietta U. Frey, Marina Mikhaylova, Michael R. Kreutz

The authors would like to add additional funding. When Drs. Suhel Parvez and Julietta U. Frey were added to the author byline, their support from DFG and Alexander-von-Humboldt Foundation should have also been included in the financial disclosure for this article. The Funding section should read: "This work was supported by NSFC (30870795, 30970920) and Scientific Innovation Projects from Fudan Univ. to TB and by the DFG (SFB779, TPB4 and TPB8 to JUF and MRK), the CBBS / Land Saxony-Anhalt / EU (C1-TP4 to MRK), DIP grant, the DZNE (to MRK), and the Schram Foundation (to MRK). SP was supported by the Alexander-von-Humboldt Foundation. The funders had no role in study design, data collection and analysis, decision to publish, or preparation of the manuscript."

